# Gene therapy for neurotransmitter‐related disorders

**DOI:** 10.1002/jimd.12697

**Published:** 2024-01-14

**Authors:** Wing Sum Chu, Joanne Ng, Simon N. Waddington, Manju A. Kurian

**Affiliations:** ^1^ Gene Transfer Technology Group, EGA Institute for Women's Health University College London London UK; ^2^ Genetic Therapy Accelerator Centre, Queen Square Institute of Neurology University College London London UK; ^3^ Wits/SAMRC Antiviral Gene Therapy Research Unit, Faculty of Health Sciences University of the Witwatersrand Johannesburg South Africa; ^4^ Developmental Neurosciences, Zayed Centre for Research into Rare Disease in Children, Great Ormond Street Institute of Child Health University College London London UK; ^5^ Department of Neurology Great Ormond Street Hospital for Children London UK

**Keywords:** AADC deficiency, DTDS, Gene therapy, inborn errors of neurotransmission, neurotransmitter disease

## Abstract

Inborn errors of neurotransmitter (NT) metabolism are a group of rare, heterogenous diseases with predominant neurological features, such as movement disorders, autonomic dysfunction, and developmental delay. Clinical overlap with other disorders has led to delayed diagnosis and treatment, and some conditions are refractory to oral pharmacotherapies. Gene therapies have been developed and translated to clinics for paediatric inborn errors of metabolism, with 38 interventional clinical trials ongoing to date. Furthermore, efforts in restoring dopamine synthesis and neurotransmission through viral gene therapy have been developed for Parkinson's disease. Along with the recent European Medicines Agency (EMA) and Medicines and Healthcare Products Regulatory Agency (MHRA) approval of an AAV2 gene supplementation therapy for AADC deficiency, promising efficacy and safety profiles can be achieved in this group of diseases. In this review, we present preclinical and clinical advances to address NT‐related diseases, and summarise potential challenges that require careful considerations for NT gene therapy studies.

## 1 INTRODUCTION

Neurotransmitters (NT) are a diverse group of chemical messengers, including the mainly inhibitory aminoacidergic (γ‐aminobutyric acid [GABA] and glycine), excitatory aminoacidergic (aspartate and glutamate), and monoaminergic (adrenaline, noradrenaline, dopamine [DA] and serotonin [5‐HT]) systems.^1^ NT are generally synthesised, stored, and released from pre‐synaptic neurons, diffuse across the synaptic cleft to bind to post‐synaptic receptors, and finally transported or enzymatically degraded for termination of neurotransmission.^1^ Inborn errors of NT metabolism (IEM‐NT) are a rare group of conditions due to defects in NT synthesis, metabolism or reuptake.^2^, ^3^ For the purpose of this review, we focus on IEM‐NT as defined by the International Working Group on Neurotransmitter‐related Disorders.^2^ However, many other disorders can also impair NT homeostasis with abnormal cerebrospinal fluid (CSF) NT profiles, including mitochondrial diseases, channelopathies and disorders of sphingolipid and cholesterol synthesis.^4^

**FIGURE 1:**
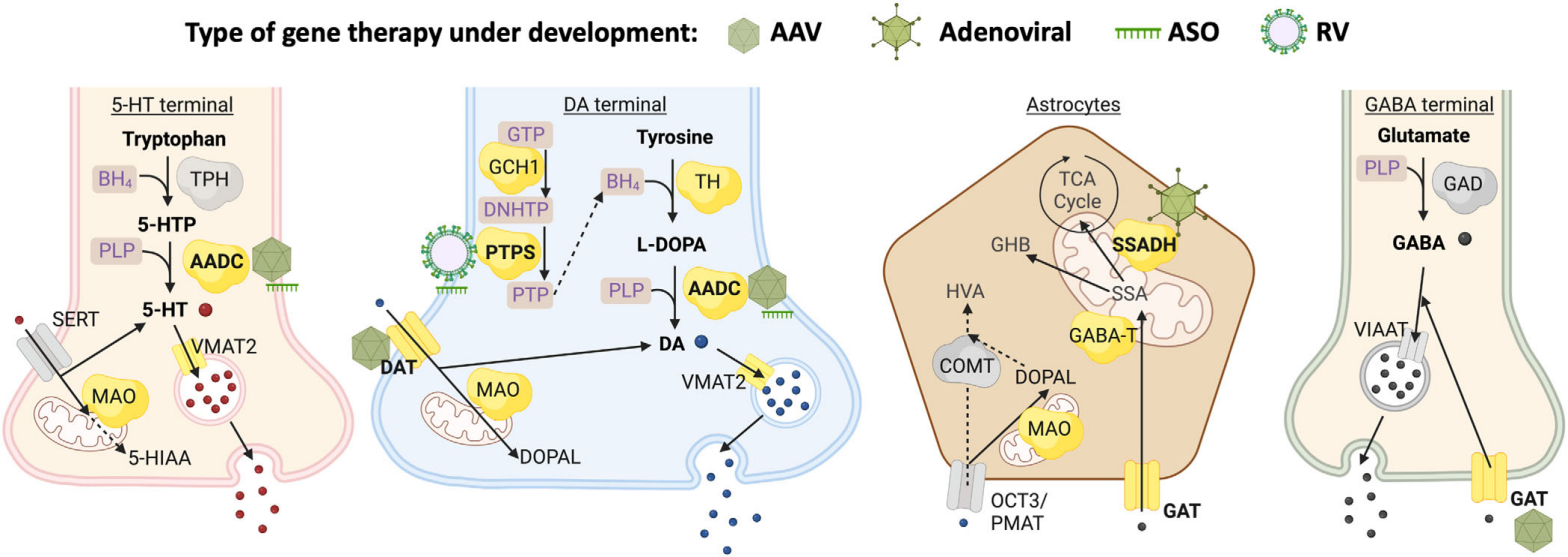
Selected pre‐synaptic neurotransmitter metabolic pathway with gene therapies in clinic and under development. Proteins known to be involved in IEM‐NT are coloured yellow; those with preclinical and clinical gene therapy investigations are in bold, and type of strategies are indicated by respective icons. Dashed lines indicate intermediate metabolic steps not shown; drawings not to scale. Created with BioRender.com. 5‐HIAA, 5‐hydroxyindoleacetic acid; 5‐HT, serotonin; 5‐HTP, 5‐hydroxytryptophan; AADC, aromatic l‐amino acid decarboxylase; AAV, adeno‐associated virus; ASO, antisense oligonucleotide; BH4, tetrahydrobiopterin; COMT, catechol‐*O* methyl transferase; DA, dopamine; DAT, dopamine transporter; DBH, dopamine β‐hydroxylase; NHTP, dihydroneopterin triphosphate; DOPAL, 3,4‐dihydroxyphenylacetaldehyde; GABA, γ‐aminobutyric acid; GABA‐T, GABA transaminase; GAD, glutamic acid decarboxylase; GAT, GABA transporter; GHB, gamma‐hydroxybutyric acid; GTP, guanosine‐5′‐triphosphate; HVA, homovanillic acid; l‐DOPA, levodopa; MAO, monoamine oxidase; OCT3/PMAT, organic cation transporter 3/plasma membrane monoamine transporter; PLP, pyridoxal phosphate; PTP, 6‐pyruvoyltetrahydropterin; PTPS, 6‐pyruvoyltetrahydropterin synthase; RV, retroviral; SSA, succinic semialdehyde; SSADH, succinic semialdehyde dehydrogenase; SERT, serotonin transporter; TCA, tricarboxylic acid; TH, tyrosine hydroxylase; TPH, tryptophan hydroxylase; VIAAT, vesicular inhibitory amino acid transporter; VMAT2, vesicular monoamine transporter 2.

The majority of IEM‐NT affects monoamine synthesis, metabolism, and reuptake, namely DA and 5‐HT (Figure 1). DA is synthesised from l‐tyrosine in two steps: to levodopa (l‐DOPA), by rate‐limiting tyrosine hydroxylase (TH) with essential co‐factor tetrahydrobiopterin (BH_4_); then by aromatic l‐amino acid decarboxylase (AADC) and co‐factor pyridoxal 5‐phosphate (PLP; active form of vitamin B_6_).^5^ DA can also be converted into noradrenaline by dopamine β‐hydroxylase. Monoamines are then packaged into vesicles by vesicular monoamine transporter‐2 (VMAT2), a neurotransmitter transporter (NTT), for subsequent Ca^2+^‐mediated docking and fusion to release into the synapse. Dopamine transporter (DAT), a plasma membrane NTT, reuptakes DA from synaptic cleft to spatiotemporally regulate synaptic DA neurotransmission.^6^ One key monoamine degradation pathway is oxidative deamination by monoamine oxidase A.^5^ Pathogenic variants have been identified in genes encoding enzymes and transporter proteins mentioned above.^7^, ^8^ Due to shared metabolic pathways between DA and 5‐HT, 5‐HT levels can be affected in pterin defects, AADC deficiency (AADCD) and VMAT2 deficiency.

Patients with IEM‐NT present with a number of common clinical features, though often with variable age of onset, severity and treatment response.^7^, ^8^ For monoamine NT disorders, the spectrum includes neonatal hypotonia, movement disorders such as dystonia, parkinsonism, eye movement disorders including oculogyric crisis (OGC), autonomic dysfunction, and developmental delay.^9^, ^10^, ^11^ Brain magnetic resonance imaging (MRI) abnormalities are also observed but are non‐specific, including reduced brain volume, delayed myelination and watershed area changes.^12^, ^13^ Previously, a timely and accurate diagnosis was a challenge due to the need for specialist diagnostic investigations, for instance, CSF NT analysis. Clinical symptoms may also mimic a range of neurological disorders (such as hypoxic ischaemic encephalopathy and cerebral palsy), resulting in misdiagnosis and diagnostic delay.^7^, ^8^ The increasing availability of exome and genome sequencing techniques has accelerated diagnosis for many rare diseases patients and has changed how children with IEM‐NTs are now identified; genetic testing now often precedes and sometimes avoids the need for CSF NT analysis.^14^

Depending on the underlying disorder, treatment approaches include use of co‐factors (e.g., sapropterin dihydrochloride, pyridoxine) and/or NT precursors (e.g., l‐DOPA, 5‐hydroxytryptophan [5‐HTP]) supplementation.^9^, ^15^, ^16^ Treatment responses vary between IEM‐NT, with some being curative, such as low dose l‐DOPA with dopa decarboxylase (DDC) inhibitor in autosomal dominant GCH1 deficiency, some with variable responses like in AADCD and TH deficiency (THD), and others being medically refractory, such as dopamine transporter deficiency syndrome (DTDS).^15^, ^16^, ^17^, ^18^ Moreover, in IEM‐NT that are treatable, including some BH_4_ disorders, symptom resolution and quality of life are often associated with early treatment initiation.^19^ As such, novel therapeutic approaches like gene therapy are promising to address underlying gene defects in drug‐resistant forms of IEM‐NT.

Gene therapy aims to deliver genetic materials by viral or non‐viral carriers, known as vectors, to address disease‐specific cellular dysfunctions.^20^ Current European Medicines Agency (EMA) and Medicines and Healthcare Products Regulatory Agency (MHRA)‐approved in vivo neurological gene therapy, namely Zolgensma® (onasemnogene abeparvovec) for Spinal Muscular Atrophy (SMA) and Upstaza™ (eladocagene exuparvovec) for AADCD, utilise viral vectors called adeno‐associated virus (AAV). In this review, we will mainly focus on AAV‐based NT gene therapies.

Wild‐type (WT) AAV is a replication‐deficient parvovirus with a 4.7 kilobases (kb) single‐stranded (ss) DNA genome; recombinant AAV vectors (rAAV) retain only the inverted terminal repeats, which involve in vector packaging, transduction and transgene expression.^21^, ^22^ Self‐complementary AAV (scAAV) bypasses the rate‐limiting second strand synthesis, thereby achieving earlier and higher level of transgene expression^21^; but induces higher immune responses than ssAAV in mice.^23^, ^24^ Different combinations of AAV capsid, regulatory elements (RE) such as promoters and delivery route are adopted depending on desired expression profile.^25^ Generally, capsid serotype determines tissue tropism due to recognition of specific cell surface receptors, followed by particle internalisation, endosomal escape, uncoating and subsequent transgene transcription.^21^, ^26^ Capsid engineering, by rational design, ancestral sequence reconstruction, or directed evolution by DNA shuffling, could further enhance transduction, specificity, and CNS‐specific axonal retrograde and/or anterograde transport.^26^, ^27^ Many novel capsids are being tested in clinical trials, for example, AAV‐LK03 (liver‐tropic,^28^ for haemophilia A (NCT03003533)^29^), and Anc80 (liver, muscle and retina‐targetting,^30^ for Wilson disease [NCT04537377]). The inclusion of *cis*‐acting RE can further define transgene specificity and expression level.^21^, ^26^ Most clinical trial gene therapy constructs employ ubiquitous promoters such as cytomegalovirus (CMV) and hybrid CMV immediate‐early enhancer/chicken β‐actin promoter (CAG).^31^ This has added benefits if transgene products can be secreted for cross‐correction, for instance, in lysosomal storage diseases.^32^ Tissue‐specific promoters could potentially limit off‐target expression and related toxicities^25^; inducible,^33^ and activity‐dependent,^34^ promoters are in development but have not reached clinical application. Recent rodent and non‐human primates (NHP) studies indicate capsid‐promoters interactions may impact cell‐type selectivity as well.^35^, ^36^ Other RE, including intron, polyadenylation (polyA) signal, and woodchuck hepatitis virus post‐transcriptional regulatory element (WPRE), increase transgene expression level by promoting mRNA stability and/or nuclear export,^37^ whereas inserting binding sites for tissue or cell‐specific microRNAs at 3′ end can regulate transgene transcript levels,^38^ and prevented transgene overexpression in mice.^39^ Delivery route is another important consideration for the desired vector biodistribution profile and safety. For CNS diseases, direct parenchymal delivery is relatively invasive and can result in focal or broad transduction; the latter is achievable by retrograde/anterograde transport due to extensive connectivity of regions such as putamen and thalamus.^40^ Alternatively, intracerebroventricular (ICV), intra‐cisterna magna, and intrathecal (IT) injections utilise CSF flow for distribution, resulting in spinal and brain transduction with some peripheral ‘leakage’.^41^ Intravenous (IV) delivery is feasible for some serotypes (e.g., AAV9, rh8, rh10) that can cross the blood–brain barrier; however, concerns about large doses, systemic genotoxicity and immunogenicity may prompt the use of alternative routes.^42^ It is also possible to undertake dual delivery, such as intrathalamic (ITH) and IT delivery is undertaken in GM2 gangliosidosis patients.^43^

One of the first NT‐related gene therapies was for Parkinson's disease (PD), which provided important clinical evidence relevant to the development of gene therapy for IEM‐NT. PD is a neurodegenerative disorder with no disease‐modifying therapy currently available; symptomatic improvement from l‐DOPA declines as PD progresses.^44^ Different gene therapy strategies have been reviewed in detail elsewhere.^45^, ^46^ Of particular interest, intraparenchymal delivery of DA (AADC, with or without TH and guanosine triphosphate cyclohydrolase 1 [GTPCH, rate‐limiting of BH_4_ synthesis]),^47^, ^48^, ^49^, ^50^, ^51^, ^52^, ^53^, ^54^ and GABA (glutamic acid decarboxylase 65 and 67 [converts glutamate to GABA]),^55^, ^56^, ^57^ synthesis enzymes were generally well‐tolerated in clinical trials. Common adverse events in DA trials included treatment‐related dyskinesia and surgery‐related events. In a Phase 2 rAAV2‐AADC trial (NCT03562494), which used a higher infusion volume of 1800 μL/putamen,^58^ T2 MRI abnormalities were observed and thus placed on clinical hold, and the sponsor partnership was terminated in 2021.^59^

**FIGURE 2:**
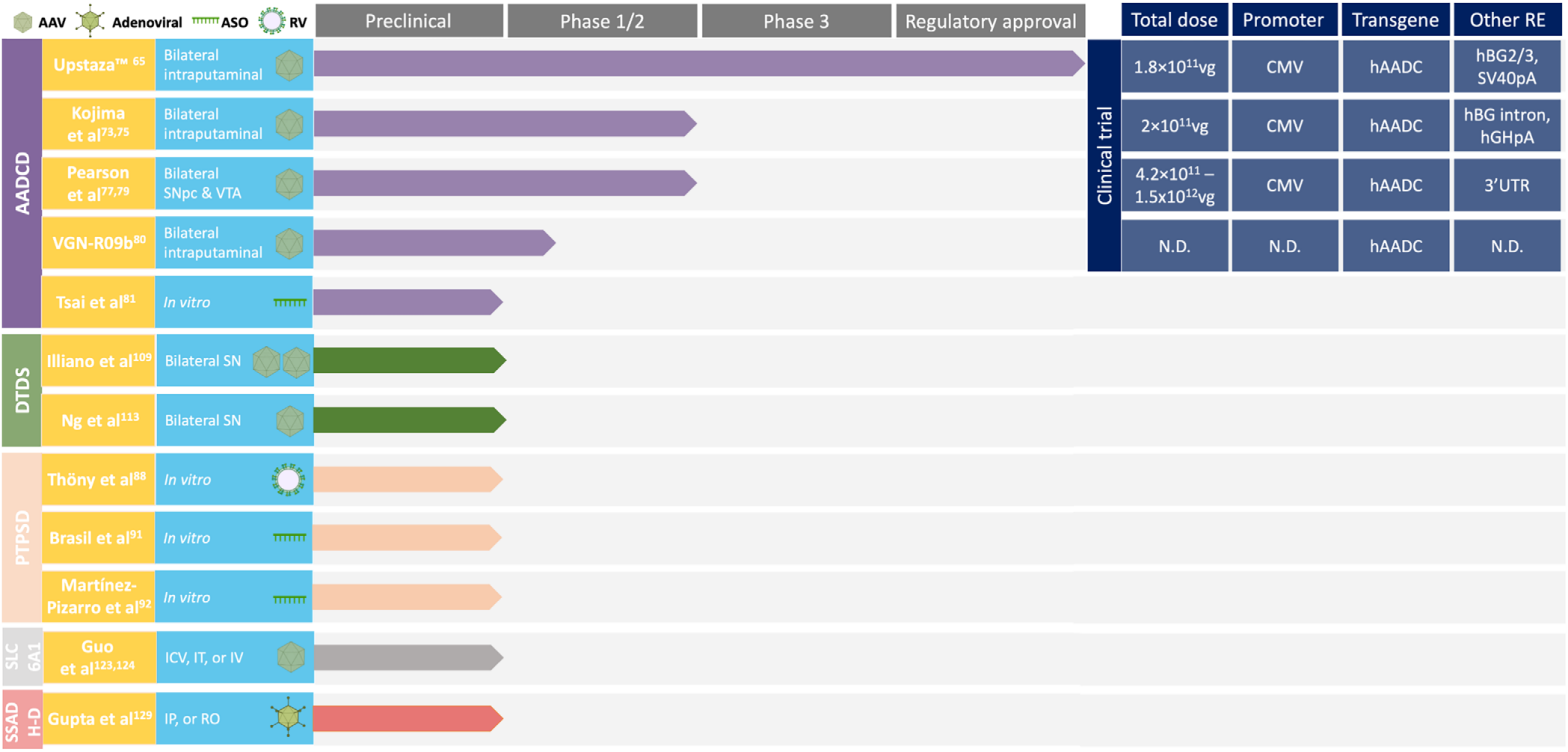
Summary of clinical and preclinical advanced therapeutics for IEM‐NT. 3′‐UTR, 3′‐untranslated region; AADCD, aromatic l‐amino acid decarboxylase deficiency; AAV, adeno‐associated virus; ASO, antisense oligonucleotide; CMV, cytomegalovirus enhancer/promoter; DTDS, dopamine transporter deficiency syndrome; hAADC, human aromatic l‐amino acid decarboxylase; hBG, human β‐globin; hBG2/3, human β‐globin partial intron 2/partial exon 3; hGHpA, human growth hormone polyadenylation signal; ICV, intracerebroventricular; IP, intraperitoneal; IT, intrathecal; IV, intravenous; N.D., not disclosed; PTPSD, pyruvoyltetrahydropterin synthase deficiency; RE, regulatory elements; RO, retro‐orbital; RV, retroviral; SLC6A1, SLC6A1‐related disorder (GABA transporter 1‐related myotonic‐astatic epilepsy); SN, substantia nigra; SNpc, substantia nigra pars compacta; SSADHD, succinic semialdehyde dehydrogenase deficiency; SV40pA, simian virus 40 early polyadenylation signal; vg, vector genomes; VTA, ventral tegmental area.

With the clinical precedents of NT gene therapies developed for PD, insights into vector design, dosage, delivery methods, safety, tolerability, and transduction of neural pathways are valuable for developing gene therapies for IEM‐NT in the clinic.^60^ The recent EMA and MHRA approvals of Upstaza™ for AADCD and recommendation by the National Institute for Health and Care Excellence (HST26) have further paved the way for the development and translation of these disease‐modifying therapies. Here, we summarise preclinical and clinical gene therapy studies for IEM‐NT (Figure 2) and discuss the potential future challenges in clinical translation.

## 2 GENE THERAPY FOR MONOAMINE NT DISORDERS

### 2.1 AADC deficiency

AADCD (OMIM #608643) is a rare neurodevelopment disorder characterised by impaired DA and 5‐HT synthesis due to biallelic mutations in the *DDC* (7p12.2) gene. Noradrenaline and adrenaline synthesis are also affected as they are downstream of DA; therefore, typical CSF markers include low DA, 5‐HT, and noradrenaline metabolites, namely homovanillic acid (HVA), 5‐hydroxyindoleacetic acid (5‐HIAA) and 3‐methoxy‐4‐hydroxyphenylglycol, along with high precursors l‐DOPA and 5‐HTP levels.^61^ A total of 120 patients have been published to date; 80% of cases are classified as severe, whereby patients typically present in the early months of life with hypotonia, OGC and developmental delay.^16^, ^62^ Autonomic dysfunction, such as excessive sweating and sleep disturbances, movement disorders, for example dystonia, and cerebral atrophy on brain MRI are also observed.^16^, ^62^, ^63^ A higher prevalence in Taiwan is attributed to a founder variant c.714+4A>T (IVS6+4A>T), and homozygous patients manifest severe phenotypes, with severe motor impairment, weight stagnation and increased risk of premature death.^64^ Existing drug treatments, including DA agonists, monoamine oxidase inhibitors, vitamin B_6_ and tonal agents such as benzodiazepines have limited benefits, and adverse effects are common.^62^

rAAV2 gene supplementation therapies targeting the putamen or midbrain have been trialled in AADCD patients. Upstaza™, approved by EMA and MHRA for severe AADCD in 18‐month‐old and above, is delivered as bilateral intraputaminal infusion (3 μL/min, 80 μL/site, 2 sites/hemisphere) at a total dose of 1.8 × 10^11^vg.^65^ The construct contains AADC cDNA, CMV promoter, human β‐globin partial intron 2/partial exon 3, and simian virus 40 (SV40) polyA. A putaminal target was chosen due to its accessibility, striatal DA deficiency, connectivity in the cortico‐basal ganglia network, and safety profiles from PD AADC trials.^66^ The compassionate use,^67^ Phase 1/2^68^ and Phase 2b^69^ trial treated a total of 21 patients (age range, 1.7–8.5 years) at 1.8 × 10^11^vg, demonstrating continuous improvement in primary endpoints of gross and fine motor development (Peabody Developmental Motor Scales–Second Edition [PDMS‐2]), significantly improved motor performance (Alberta Infant Motor Scale [AIMS]), cognitive function, language skills and AADC activity in putamen, as measured by PET ^18^F‐DOPA scans, up to 5 years post‐treatment.^69^ Milestone attainments, such as head control and sitting unassisted, were progressive, with 44% and 20% at 1 year and 75% and 67% at 5 years, respectively.^70^ Reduced OGC frequency, increased CSF HVA (but not 5‐HIAA), and improvement in weight gain, autonomic (sweating) and serotonergic (mood) symptoms were also reported 1‐year post‐treatment.^67^, ^68^, ^69^ No significant difference in PDMS‐2 was achieved with higher dosage at 2.4 × 10^11^vg in the Phase 2b study; rather, earlier treatment was significantly correlated to higher PDMS‐2 scores and improvement in white matter microstructure on MRI.^69^, ^71^ Three patients that are able to walk without assistance post‐treatment were also treated early, by 4.2 years old.^69^ Adverse events include surgical complications, pyrexia, and transient orofacial and limb dyskinesia, possibly due to early DA receptor hypersensitivity. Dyskinesia onset within 3 months of treatment, was relieved by risperidone and resolved by 10 months; severity and duration correlated with age of patients rather than dosage.^69^ Lately, motor and non‐motor improvements 1‐year post‐treatment were reported in two older patients (treated when >10 years old) with severe AADCD, with improved putaminal ^18^F‐DOPA uptake compared to baseline.^72^

Similar results were shown in a Japanese Phase 1/2 rAAV2 study also using CMV promoter, showing significant putaminal AADC activity detected by PET, improved motor functions (AIMS), and reduced OGC duration up to 2 years post‐gene therapy in severe AADCD patients.^73^ The gene therapy was administered at a higher concentration (i.e., lower infusion volume of 50 μL/site, with a similar total dose of 2 × 10^11^vg). Variable responses in CSF HVA levels and cognition were seen; transient orofacial dyskinesia and choreic movements occurred in all six patients, peaking at 2 months and diminishing by 6 months.^73^ Further analysis showed PET tyrosine tracer uptake was significantly increased in the putamen and substantia nigra (SN) post‐gene therapy, and associated with gross motor improvement via prefrontal cortico‐putaminal network restoration.^74^ Treatment of two patients with moderate AADCD, at 4 and 12 years of age, respectively, led to improvements in AIMS score, but improvement in the development quotient score was only seen in the earlier‐treated patient.^75^

Unlike in PD, midbrain structures in AADCD are not known to degenerate.^76^ Therefore, another approach is to directly deliver rAAV2‐AADC to the midbrain, specifically to the SNpc and ventral tegmental area (VTA) DA neurons, to address DA deficiency in nigrostriatal and other dopaminergic pathways.^77^ Also driven by a CMV promoter, a Phase 1 (NCT02852213) study of seven patients (aged 4–9 years old) were treated at 1.3 or 4.2 × 10^11^vg total dose by bilateral MRI‐guided convection‐enhanced delivery (50 μL in SNpc and 30 μL in VTA/hemisphere). Eighty percent coverage of both areas was obtained; ^18^F‐DOPA PET scan showed increased AADC activity in both the midbrain and striatum at 3 and 24 months, likely due to anterograde axonal transport of vector and AADC protein. Notably, complete and sustained resolution of OGC occurred within 9–33 days for 5 subjects; 6 (86%) gained head control and 4 (57%) could sit independently by 12 months. All five patients followed up till 18 months achieved clinically meaningful improvement in motor score (Gross Motor Function Measure; GMFM‐88), reduction in irritability and insomnia. Similar to the trials reported above, post‐treatment improvement in CSF HVA (but not 5‐HIAA), and transient dyskinesia in all patients 3–4 weeks after treatment were observed. Ongoing dose‐escalation study evaluated a larger volume (up to 300 μL, 1 infusion/hemisphere, in between SNpc and VTA) and dose up to 1.5 × 10^12^vg, and with a patient age range spanning from 4‐ to 27‐year‐old.^78^, ^79^

Recently, an early Phase 1 trial delivering AAV9‐AADC (VGN‐R09b) to bilateral putamen was initiated in China (NCT05765981).^80^ The trial is recruiting children between 2 and 8 years with homozygous or compound heterozygous IVS6+4A>T missense and baseline motor development <3 months. Outcome measures are adverse events and motor development at 1 year. Dosage and construct design are not available.

Finally, since the founder variant c.714+4A>T results in aberrant splicing and produces a premature stop codon from a pseudoexonic +38 cryptic splice site,^64^ an antisense oligonucleotide (ASO) strategy was tested in vitro to restore normal mRNA splicing.^81^ ASO binds to target mRNA via Watson‐Crick base pairing, and modulate pre‐mRNA splicing via steric hindrance for the recruitment of splicing factors.^82^, ^83^ The desired properties can be generated by chemical modifications of backbone, sugar moiety and/or nucleoside.^82^, ^83^ Using a phosphorodiamidate morpholino oligomer (PMO), up to 41% restoration in mRNA level was observed after 72‐h transfection in patient‐derived lymphoblastoid, with significant increase in AADC protein and 5HT levels.^81^ However, two new out‐of‐frame isoforms arouse, indicating the need to refine and/or use of combinatory ASOs to ensure appropriate splicing to minimise the risk of off‐target effects.

### 2.2 Pyruvoyltetrahydropterin synthase deficiency

Pyruvoyltetrahydropterin synthase (PTPS) is the second step of BH_4_ synthesis from GTP.^84^ It is the most common BH_4_ disorder identified by newborn screening (due to the presence of hyperphenylalaninaemia). Pyruvoyltetrahydropterin synthase deficiency (PTPSD) (OMIM #261140) has a broad phenotypic spectrum and can be biochemically characterised into mild and severe forms with reduction of HVA and 5‐HIAA, which generally requires BH_4_ and NT precursor supplementations with variable response.^15^, ^85^ One hundred ninety‐nine autosomal recessive variants of the *PTS* gene, located at 11q22.3‐23.3, have been identified, including 115 missense and 26 splicing substitutions; protein structures, particularly active site BH_4_ binding region, are known to be disrupted.^86^, ^87^ To address the genetic defect, a retroviral (RV) gene transfer and two ASO strategies have been evaluated in patients fibroblasts. In PTPSD patients, low biopterin and high neopterin are observed; following Moloney murine leukaemia virus (MLV)‐based RV transfection, PTPS enzyme activity and a trend for pterin normalisation was observed in all three patient fibroblast lines.^88^ However, a potential translation challenge is that MLV vectors are inefficient in transducing slowly or non‐dividing cells, as well as concerns about insertional mutagenesis.^89^ Splice site variants can lead to aberrant intron inclusion (pseudoexons), as mediated via mechanisms such as novel 5′ or 3′ splice site, and influencing splice enhancer/silencer.^90^ Therefore, ASO blockage of these cryptic splice sites could facilitate the expression of more functional proteins.^83^ The two approaches utilised different ASO chemistries, specifically PMO^91^ and 2′‐*O*‐methyl phosphorothioate,^92^ to block the recruitment of splicing factors for pathogenic pseudoexon production. Splice site‐targeted ASOs were able to rescue three deep intronic mutations, which normally result in extra amino acid insertions, premature termination, and frameshift,^93^ in patients' fibroblasts on mRNA and protein levels.^91^ A further study on another intron 2 variant provided evidence that the same ASO can be used in variants affecting the same region, such as a common splice site or regulatory binding motifs.^92^

## 3 NT TRANSPORT

### 3.1 Dopamine transporter deficiency syndrome

DAT is vital in regulating DA neurotransmission by reuptake of extracellular DA into midbrain dopaminergic (mDA) neurons, thereby regulating mDA excitability.^94^ DAT belongs to the solute carrier (SLC) 6 family, and co‐transports Na^+^ and Cl^−^ along electrochemical gradients, to drive DA translocation against a concentration gradient via alternating access.^95^, ^96^ Gene variants have been implicated in PD, attention deficit hyperactivity disorder, autism and neuropsychiatric disorders.^97^, ^98^, ^99^ DTDS (OMIM #613135) results from biallelic loss‐of‐function mutations in *SLC6A3*. Classical DTDS is an infantile‐onset, progressive motor disorder, with symptoms including hyperkinesia, orolingual dyskinesia, parkinsonism‐dystonia and ocular flutter; atypical juvenile and adult‐onset forms are also reported with a less aggressive disease course, and associated with higher residual DAT function.^18^, ^100^, ^101^ Evidence of neurodegeneration by DAT‐single‐photon emission computed tomography (SPECT) was observed in two atypical adult patients.^102^, ^103^ There is one adult case where autism spectrum disorder and PD are associated with a dominant‐negative variant identified via exome sequencing.^102^ In DTDS, loss of DAT function results in extraneuronal DA accumulation, leading to elevated CSF HVA but normal 5‐HIAA.^101^ Little benefit from pharmacological treatments is observed in patients^101^; whilst pharmacochaperones could potentially correct mutation‐related protein folding defects and DAT surface expression,^104^ such approaches are mutation‐specific, as opposed to a gene supplementation strategy, which could treat a broader spectrum of DTDS patients.

DAT‐knockout (KO) mice recapitulate major human DTDS motor features, including progressive motor deficits, dyskinesia, tremors and metabolic profiles of high HVA and low DA; shortened lifespan with parkinsonism and reduced TH levels were also found in these mice.^105^, ^106^, ^107^ A dual rAAV2/10 strategy, utilising Cre/LoxP system (FLEX switch^108^) for specific DA neuronal expression, was evaluated in adult post‐natal (P70) DAT‐KO mice.^109^ Briefly, the GOI (mouse *Slc6a3* or reporter gene), in inverted antisense orientation, was put into a double‐inverted open reading frame containing two pairs of reciprocally orientated LoxP sites, driven by a ubiquitous (CMV) promoter and packaged as the first rAAV. The second rAAV contains a target cell/ tissue‐specific promoter (midbrain DA neuronal promoter, rat TH^110^) to deliver Cre‐recombinase, which can recognise the LoxP sites and inverse the GOI sequence, therefore allowing transgene expression.^109^, ^111^ Bilateral stereotactic SN injections of the two vectors, approximately at 5 × 10^9^vg each, resulted in DAT expression and significant improvements in striatal extracellular DA levels and restoration of TH in the dorsal striatum.^109^ Further behavioural, survival, tissue and metabolite analyses showed significant improvements compared to reporter gene‐treated KO, and in some, phenotype restoration was comparable to wild‐type (WT). Whilst this approach has provided great proof‐of‐concept for rAAV‐DAT gene therapy, the use of a murine transcript, P1 bacteriophage‐derived Cre/LoxP, and potential Cre‐related neurotoxicity^112^ will limit clinical translation.

Our group previously showed in mDA neurons derived from DTDS patient fibroblasts, DAT dysfunction is associated with TNF‐α‐mediated apoptotic neurodegeneration, which was not seen in age‐matched and isogenic lines.^113^ Lentiviral transduction of human *SLC6A3* rescued DA uptake and prevented neurodegeneration. For proof‐of‐principle in vivo investigation, rAAV9‐SLC6A3 driven under truncated human synapsin 1 (hSyn1) promoter was injected neonatally by ICV at 2 × 10^10^ and 2 × 10^11^vg/pup in DAT‐KO mice. Compared to untreated DAT‐KO, low dose increased 1‐year survival from 41% to 100%, restored body weight, locomotor behaviour, DA and HVA profiles, and striatal neuronal firing. Widespread, rostrocaudal transduction was observed, with no dose‐dependent increase in target mDA neurons; rather, brain‐wide overexpression in high‐dose animals led to adverse events requiring euthanasia in 50%, cortical reactive astrogliosis, neuronal loss and vacuolation. Therefore, to combat these observed off‐target effects, a more restricted rAAV2 was used to treat symptomatic DAT‐KO at 4 weeks; 3‐log dose‐ranging (2 × 10^8^–10^10^vg/mouse) showed a dose‐dependent increase in mDA neurons, human DAT protein, mRNA, and vector genome copy in the midbrain, with evidence of anterograde transport to the striatum. All doses rescued foot fault to WT level, with high dosage 2 × 10^10^ demonstrating open field and vertical pole descent time equivalent to WT. No neuropathology was observed in with these dosages, delivery route and capsid. Gene therapy for DTDS has recently been granted orphan drug designation by EMA and rare paediatric disease designation by FDA, with a Phase 1/2/3 clinical trial currently being planned.^114^

### 3.2 SLC6A1‐related disorder (GABA transporter 1‐related myotonic‐astatic epilepsy)

GABA transporter (GAT)‐1 and ‐3 are the major GATs which are widely expressed in different cell types throughout the brain; GAT‐1, encoded by *SLC6A1* on chromosome 3, is mainly localised to axonal terminals of GABAergic neurons and astrocytes.^115^, ^116^ Myoclonic astatic epilepsy (OMIM #616421), caused by haploinsufficiency of *SLC6A1*, is a developmental and epileptic encephalopathy, associated with myoclonic–astatic and absence seizures, intellectual disability, autism spectrum disorder, and hypotonia in early years of life.^117^, ^118^ Around 120 patients have been published.^117^ Loss‐of‐function *SLC6A1* variants lead to impaired protein trafficking and GABA transport activity; increased extracellular and reduced intracellular GABA levels are postulated to cause tonic and phasic inhibitions.^119^, ^120^ Given GABA's role in brain development from the early embryonic stage until maturity,^121^ restoration of GAT‐1 expression with gene therapy may be beneficial over current symptomatic treatments.

TSHA‐103, a scAAV9 driving codon‐optimised copy of human *SLC6A1*, is currently in preclinical development by Taysha Gene Therapies.^122^ In a homozygous *SLC6A1*‐KO mice model, neonatal ICV delivery at 3 × 10^11^vg/pup of two constructs driven by either a ubiquitous (JeT) or a neuronal‐specific (MeP) promoter significantly reduced seizure burden. Behavioural improvements were only noted in the neuronal‐specific version. Furthermore, adverse effects were observed, including higher mortality with the ubiquitous promoter.^123^ For clinical translation, older mice were treated IT at 7–7.5 × 10^11^vg/mouse, with lower cortical expression than neonatal ICV but was well‐tolerated for up to 1 year. However, only modest efficacy in behavioural tests was observed when treated at P7–10, with no improvement in the P28–35 group.^123^ To evaluate whether therapeutic efficacy is linked to transduction efficiency or earlier intervention, AAV‐PHP.eB, a neurotropic variant of AAV9, was used for IV injection in P23 mice.^124^ Seizure reduction was not achieved at any of the three doses (ranging from 2 × 10^10^ to 1 × 10^12^vg/mouse). This suggests both a narrow therapeutic window and that further expression optimisation is required.

## 4 NT DEGRADATION

### 4.1 Succinic semialdehyde dehydrogenase deficiency

Succinic semialdehyde dehydrogenase (SSADH) is a systemically expressed mitochondrial enzyme, with major expression in the brain and liver.^125^ In healthy individuals, synaptic GABA is transported into astrocytes by GAT, converted to succinic semialdehyde by GABA transaminase (GABA‐T), then oxidised in tandem by SSADH to succinate, a substrate of the TCA cycle.^125^ In SSADHD (OMIM #271980), biallelic loss‐of‐function mutations in the encoding *ALDH5A1* (6p22.3) gene result in the cytosolic conversion of succinic semialdehyde to γ‐hydroxybutyrate (GHB), a neuromodulator with neurotoxic properties, instead.^126^ Excess accumulations of GABA, GHB and other GABA metabolites are found in brain and body fluids, but the exact pathophysiology remains to be elucidated.^125^ Clinical symptoms, including hypotonia, developmental delay, epilepsy, and behavioural and sleep disturbances, manifest in early childhood^126^; age‐dependent association in symptom severity and seizure types have been described.^127^ Post‐mortem study of one patient also found amino acid, phospholipid, and NT disturbances.^128^ No effective treatment currently exists to address the underlying deficiency and downstream metabolic sequelae.

As the liver also expresses high levels of SSADH, and central and peripheral GHB rapidly equilibrates,^125^ a liver‐directed adenoviral approach was evaluated in a mouse SSADH‐KO model.^129^ These mice recapitulate metabolic features observed in humans but have more severe phenotypes, with reduced survival up to P26 due to status epilepticus.^130^ Using a first‐generation E1‐deleted adenovirus, human SSADH cDNA was delivered under a potent Rous sarcoma virus promoter intraperitoneally (IP) at P10 at three doses (4.5 × 10^8^, 1 × 10^10^, 1 × 10^11^ viral particles [vp]), and retro‐orbitally (RO) at P13 with 1 × 10^11^vp.^129^ Extension in survival was observed in all treatment groups, highest by 39.3% in the 4.5 × 10^8^ IP group. For tissue analysis, different sets of mice were injected and tissues were collected at different timepoints; at 72 h post‐injection, higher hepatic SSADH enzymatic activity was achieved by RO (maximum ~20%) than IP. RO‐treated also had GHB reduction in the liver, brain, kidney and serum, but restricted to the liver only for IP. In treated mice that survived beyond 1 month, mRNA but not SSADH enzyme activity was detected. Neonatal (P0) IP injections reduced liver GHB for up to 8 days, but was accompanied by unexplained rebound elevation in the brain. Overall, the vector appeared to be safe, but transient expression secondary to immunogenicity, dividing nature of hepatocytes, and hepatic‐only targeting may have limited its effectiveness, all factors that need to be addressed for future clinical translation.

## 5 DISCUSSION

Effective management of rare diseases, including IEM, requires accurate diagnoses and disease‐modifying treatments. For IEM‐NT, only a handful of disorders are identified through newborn screening and there can often be diagnostic delay. The clinical responses to existing small molecule pharmacotherapies are variable from disease to disease, with some evidence showing that earlier treatment initiation is associated with better outcomes for some IEM.^8^, ^19^ Advances in genome/exome sequencing, transcriptomics, and other complementary diagnostic techniques has significantly improved the diagnostic rate of rare diseases patients.^14^ Other avenues include building on existing newborn screening programmes, which are either already adopted or being currently validated for AADCD. In Taiwan, Italy, Germany and Brazil, the diagnostic utility of detecting high levels of 3‐*O*‐methyldopa in dried blood spots via mass spectrometry is under evaluation.^131^, ^132^, ^133^, ^134^ In terms of treatments, gene therapy, both preclinically and clinically, is proving to be a promising disease‐modifying approach for many IEM.^135^, ^136^ Other technologies, such as gene‐editing, utilises machinery such as zinc‐finger nucleases and CRISPR‐Cas (clustered regularly interspaced short palindromic repeat‐Cas‐associated nucleases), delivered in vivo as proteins in nanoparticle delivery systems or nucleic acid in AAV, are being evaluated preclinically and clinically for some IEM conditions.^137^, ^138^

As the majority of IEM‐NT are monogenic and autosomal recessive, AAV gene supplementation therapy is an attractive, one‐off, disease‐modifying treatment for many of these rare diseases. This offers some advantages over ASOs that require repeated direct administration into CNS, and avoids the need to tailor guide RNAs for each pathogenic mutation in gene editing. Safety and tolerability of NT‐related gene therapies in humans have been evaluated with PD, and efficacy has been clearly shown in AADCD by positive trial outcomes culminating for putaminal‐delivered^67^, ^68^, ^69^, ^73^ and midbrain‐delivered^77^ gene therapy, with Upstaza™ receiving EMA and MHRA approval in 2022.

With gene therapies now reaching the clinical arena, there are new aspects to consider as we learn more about AAV gene therapies, for instance, new safety concerns, optimal therapeutic window, ease of global access to treatment, and how to navigate multiple treatment options for each IEM‐NT. Specific safety concerns of CNS AAV gene therapies regarding dorsal root ganglion toxicity, with neuronal degeneration and axonopathy associated with a variety of rAAV capsids and promoters, having been attributed to transgene overexpression in NHP.^139^, ^140^ Dose‐associated toxicity via ITH administration, with highest‐dosed NHP at 3.2 × 10^12^vg of a rAAVrh8 vector expressing Hexα/β, showed ataxia and general weakness at 2–3 weeks and requiring euthanasia due to apathy by 1 month.^141^ To date, the clinical relevance of DRG toxicity in humans has yet to be elucidated, with no treatment‐related DRG toxicity reported in Zolgensma®'s trials and global access programme.^142^, ^143^, ^144^ Moreover, tight control of NT systems may imply a narrow therapeutic dosing window (i.e., between efficacy and toxicity), as seen in our DTDS preclinical gene therapy study, whereby a 10‐fold increase in dose resulted in unexpected weight loss and motor disturbances related to off‐target effects.^113^ Careful dose calibration in achieving efficacy and minimal toxicity can be challenging, given the irreversibility of AAV gene therapy, the long persistence of AAV genome (up to 15 years in NHP for PD^145^), and potential epigenetic silencing of rAAV genomes in vitro^146^, with relevance in human patients yet to be determined. As described previously, strategies to finetune expression might include optimisation of delivery routes, capsid, promoters, and/or RE.^27^

Specific to IEM‐NT disorders, we must recognise that NTs are vital in neurodevelopment from the embryonic stages,^147^ so one consideration is how to diagnose patients at the earliest opportunity, and ensure that treatment is administered within the optimal treatment window for maximal therapeutic efficacy. In BH_4_ deficiencies, it is already recognised that earlier treatment is linked to better cognitive outcome, but it is unclear whether the developmental trajectory can be completely restored to normal.^19^, ^148^ Similarly, AADC gene therapy trials suggest younger patients achieved better motor scores, and brain structure improvements. Notably, all patients who gained the ability to walk were treated by ~4 years of age.^69^, ^71^ In THD, mild–moderate cognitive delay is observed in a significant portion of patients who otherwise have a beneficial response to l‐DOPA.^17^ Abnormalities in neuronal and synaptic development were observed in a miscarried foetus with THD as early as 16 weeks gestation.^149^ In addition, for IEM‐NT that affects more than one NT, such as the case for AADC, careful evaluations are needed in designing the target(s) of the gene therapy product to achieve maximum patient benefits. 5‐HT is produced in raphe nuclei throughout the brainstem; current strategies targeting the putamen or midbrain might not be directly addressing 5‐HT deficiency, as CSF 5‐HT metabolite (5‐HIAA) was not significantly increased after delivery.^69^, ^77^ Although significant symptomatic improvements were seen in the carer‐reported questionnaire,^69^, ^77^ implications in the longer‐term management of these patients are still unclear. Moreover, interactions within different components of the NT synthesis pathway can be altered in disease state, with the exact mechanisms and long‐term implications not fully understood. One example is different BH_4_ deficiency murine models, namely sepiapterin reductase,^150^ PTPS,^151^ and GCH1,^152^ where reduction in TH protein levels, particularly in striatum, were observed, impacting foetal DA circuit development.^153^ Normal TH levels, therefore, appear critical for psychomotor function and DA system maturation.^154^ It may be that gene therapies for IEM‐NT would be most efficacious in younger patients, providing a case for earlier diagnosis and potential foetal/newborn screening for IEM‐NT. More extensive natural history studies of these rare disorders will, therefore, be invaluable when developing new therapies to better inform about the timing of gene delivery.

Another important consideration is accessibility for patients; the list price for Upstaza™ is £3 million (~€3.4 million),^155^ whereas Zolgensma® for SMA is €1.95 million. Discount negotiations, reimbursement schemes, and cost‐effectiveness calculations measured by quality‐adjusted life year vary by country,^156^ and so regional differences are likely expected. Part of the cost is attributed to the complexities of AAV GMP manufacturing and downstream processing.^157^ Moreover, the need for pre‐ and post‐administration monitoring and establishment of long‐term efficacy and safety profiles (10 years required for Upstaza™),^65^ likely means only specialised hospital sites can provide these therapies, potentially limiting broader access.

In the rapidly growing space of advanced therapies, multiple strategies will be approved and licenced, for the same disease, such as in the case of SMA. For AADCD, three gene therapy clinical trials and Upstaza™ utilise different AAV gene supplementation strategies. However, the clinical impact of the different brain targeting, dosage, capsids, and construct designs (with differing RE) is not yet clear. Putaminally delivered DA restoration in the prefrontal cortico‐putaminal network was found to be important for motor improvement,^66^ and directly targeting the mDA neurons may improve or restore physiological DA homeostasis, and potentially protect from DA‐induced oxidative stress, that may occur with putaminal dopamine synthesis. Moreover, expression profiles vary with different capsids, promoters, and RE. AAV2 shows properties of anterograde transport with predominant neuronal transduction, whilst AAV9 shows both anterograde and retrograde transport, and neuronal and glial transduction.^158^ The AADCD clinical trials (where disclosed) have used ubiquitous CMV promoters, yet no head‐to‐head in vivo studies have been performed to compare construct expression efficiency or other differences (Figure 2), which may result in variable mRNA levels and stability. From a clinical perspective, different inclusion ages (Upstaza™ 18 months and over, Kojima et al.^73^, ^75^ and Pearson et al.^77^, ^79^ 4 years and over, VGN‐R09b^80^ 2–8 years old), variable baseline disease severity, and relatively small patient groups may make direct comparison difficult, as well as the use of different outcome measures (e.g., PDMS‐2 vs. GMFM‐88). Over time, it would be of utmost patient benefit to have an international, standardised approach from pre‐screening to post‐gene therapy surveillance, using appropriate disease‐specific registries, to consolidate clinical data, allowing for thorough treatment evaluations, especially in rare diseases like IEM‐NT.^159^

Adverse effects of gene therapy for IEM‐NT would likely depend on the NT pathway(s) targeted. For example, for most AADCD patients, transient dyskinesia is expected around 4 weeks post‐therapy. This is likely a result of AAV‐driven AADC‐related DA production, leading to potential hypersensitivity of DA receptor, and should resolve as DA homeostasis stabilises.^69^, ^73^ Management of these dyskinesias depends on trial protocol or local guidelines and should be overseen by a clinician with expertise in this area. Generally, treatment should be initiated if the movements interfere with routine care, function, comfort, sitting or sleeping. Any DA agonists or monoamine oxide inhibitors may be tapered and weaned off, as necessary.^77^, ^159^ Agents such as amantadine, benzodiazepines, gabapentin, clonidine or even tetrabenazine may be considered for the short‐term management of this transient dyskinesia.^72^, ^79^ Some patients will need more intensive medical management within intensive care or high dependency.

Overall, with the rapid innovations in vector technology and a better understanding of pathophysiology and molecular diagnosis, gene therapy for IEM‐NT is a growing field that could soon become a clinical reality for many diseases. Early diagnosis and treatment would likely be key for IEM‐NT, and accessibility would be fundamental in achieving the full potential and clinical impact of these new treatments globally.

## AUTHOR CONTRIBUTIONS

Wing Sum Chu drafted and revised the manuscript. Joanne Ng, Simon N. Waddington and Manju A. Kurian gave substantial intellectual input in manuscript critique and revision.

## CONFLICT OF INTEREST STATEMENT

Joanne Ng is an inventor on patent application titled Gene therapy for DTDS (GB2101958.3), has consultancy agreements with Albion Venture Capital, sponsored research agreements with Synpromics/Askbio Europe, Rocket Pharma, Helex Bio and Bloomsbury Genetic Therapies and holds equity in Bloomsbury Genetic Therapies. Simon N. Waddington is an inventor on patent application titled Gene therapy for DTDS (GB2101958.3), holds consultancy agreements with Biormarin and Albion Capital, has previous or existing consultancy agreements with ONO Pharmaceuticals, Synpromics Ltd., Reliance Biosciences, Codiak Biosciences, Takeda Pharmaceutical Company and LivaNova Plc., has sponsored research agreements with Synpromics/Askbio Europe Rocket Pharma, Helex Bio and is founder of Bloomsbury Genetic Therapies. Manju A. Kurian is an inventor on patent application titled Gene therapy for DTDS (GB2101958.3), was sponsored by Agilis to attend the AADC Deficiency International Advisory Board (2018), received honoraria for speaking at a PTC sponsored symposium (2023), and is founder of Bloomsbury Genetic Therapies. Wing Sum Chu declares no conflict of interest.

## ETHICS STATEMENT

This article does not contain any studies with human or animal subjects performed by any of the authors.

## Data Availability

Data sharing is not applicable to this article as no new data were created or analysed in this study.
